# Particularity of Cerebral Microcirculatory Changes in Dementia Patients with Alzheimer’s Disease Compared to Other Cerebrovascular Diseases

**DOI:** 10.1002/alz.087896

**Published:** 2025-01-03

**Authors:** Ivan V Maksimovich

**Affiliations:** ^1^ Clinic of Cardiovascular Diseases named after Most Holy John Tobolsky, Moscow, Moscow Russia

## Abstract

**Background:**

Dementia aggravates most cerebrovascular lesions, which requires differentiating the developed microcirculatory changes when making a diagnosis.

We consider the features of cerebral microcirculation disorders in Alzheimer’s disease (AD), distal cerebral atherosclerosis, Binswanger’s disease (BD), and vascular parkinsonism (VP).

**Method:**

The study included 1024 patients who underwent: assessment of CDR, TDR, MMSE, cerebral MRI, MRA, CT, MSCTA, scintigraphy (SG), rheoencephalography (REG), cerebral multi‐gated angiography (MUGA).

Patients were divided into:

• Test group ‐ 93 people with AD aged 34‐80 (average age 67.5): men ‐ 32 (34.40%), women ‐ 61 (65.59%). According to dementia severity, patients were divided into (TDR‐0) ‐ 10, (TDR‐1) ‐ 29, (TDR‐2) ‐ 36, (TDR‐3) ‐ 18 people;

• Control group ‐ 931 people aged 29‐81 (average age 78): men ‐ 687 (73.79%), women ‐ 244 (26.21%) with distal cerebral atherosclerosis ‐ 842 (90.44%), BD ‐ 27 (2.9%), VP – 62 (6.66%) people. According to dementia severity, patients were divided into unnoticed dementia ‐ 570, dementia at level (CDR‐1) – 333, (CDR‐2) ‐ 28 people.

**Result:**

In all test group AD patients, changes in angioarchitecture and microcirculation manifest themselves in:

• absence of atherosclerotic changes in intracerebral arteries,

• increased tortuosity of intracranial arteries’ distal parts,

• the capillary bed reduction in the temporal and frontoparietal regions,

• development of multiple, large arteriovenous shunts with early venous discharge of arterial blood in the same sections,

• abnormal, intracerebral venous trunks development,

• development of intracerebral venous blood stagnation,

These changes are called “Dyscirculatory Angiopathy of Alzhemer’s type” (DAAT).

In control group patients, changes in angioarchitecture and microcirculation manifest themselves in:

• multiple deposits of calcium salts and atherosclerotic changes in intracerebral arteries,

• local decrease in cerebral capillary blood flow in the same sections,

• development of local small intracerebral arteriovenous shunts,

• absence of marked intracerebral stagnation of venous blood.

**Conclusion:**

Dyscirculatory angiopathy of Alzheimer’s type (DAAT), regardless of dementia severity, is a cerebrovascular and microcirculatory brain lesion specific to AD, not associated with the atherosclerotic process.

In patients with distal intracerebral atherosclerosis, BD and VP, there are no specific changes similar to DAAT. Vascular and microcirculatory changes are atherosclerotic in nature.

